# WDR91, an endosomal maturation protein, promotes antisense oligonucleotide activity

**DOI:** 10.1016/j.omtn.2026.102915

**Published:** 2026-04-04

**Authors:** Suxiang Chen, Tong Zheng, Dunhui Li

**Affiliations:** 1 Personalised Medicine Centre, Health Futures Institute, Murdoch University, Murdoch, WA 6150, Australia; 2 Department of Kidney Transplantation, The Third Affiliated Hospital of Sun Yat-sen University, Tianhe District, Guangzhou, Guangdong Province 510630, China; 3 Stephen & Denise Adams Center for Parkinson’s Disease Research, Yale School of Medicine, New Haven, CT 06510, USA; 4 Department of Neurology, Yale School of Medicine, New Haven, CT 06510, USA

## Main text

Antisense oligonucleotides (ASOs) have emerged as an important class of nucleic acid therapeutics that enable sequence-specific targeting of RNA. Despite substantial advances in ASO chemistry and therapeutic development, efficient intracellular delivery remains a major challenge, as only a small fraction of internalized ASOs escapes endosomal compartments to reach the cytoplasm or nucleus.^1^ In a recent study published in *Molecular Therapy – Nucleic Acids*, Menchon and colleagues used a genome-wide CRISPR screen to identify regulators of ASO activity and uncovered the endosomal maturation protein WD repeat domain 91 (WDR91) as a key promoter of ASO activity.^2^ Loss of WDR91 markedly reduced ASO activity, suggesting that endosomal maturation process may influence oligonucleotide activity. This finding underscores the importance of intracellular trafficking pathways in determining ASO performance and raises important questions about how endosomal maturation may regulate the release of oligonucleotides from endosomal compartments.

Endosomal compartments constitute a dynamic intracellular network that governs the trafficking and sorting of internalized molecules, including ASOs.^3^ The maturation from early to late endosomes, regulated by vesicular transport proteins, determines whether internalized oligonucleotides are degraded, recycled, or released into the cytoplasm or nucleus. Understanding these pathways can provide a framework for how cellular factors modulate ASO efficacy.

CRISPR-based screening has emerged as a powerful tool for systematically identifying genes that regulate cellular phenotypes. Such screens commonly use CRISPR activation (CRISPRa) to upregulate gene expression or CRISPR knockout (CRISPR KO) to disrupt gene function, enabling unbiased identification of regulators of specific cellular processes. McMahon et al. (2023) used a CRISPRa screen to identify genes, including *GOLGA8*, that enhance ASO uptake and intracellular activity in mammalian cells.^4^ In contrast, Malong et al. (2025) performed a genome-wide CRISPR KO screen to reveal proteins at the endosome-Golgi interface that influence cellular ASO activity.^5^ Together, these studies highlight the utility of CRISPR-based screening for identifying cellular regulators of ASO activity.

In the present study, a fully tricyclo-DNA (tcDNA)-phosphorothioate (PS)-modified target site blocker (TSB) ASO, named tcT3, was used to exert anti-proliferative activity in 501Mel melanoma cells by preventing sequestration of the tumor suppressor *miR-16* by its sponge, *TYRP1* mRNA, thereby redirecting *miR-16* to its target RNAs. To identify regulators of tcT3 activity, a genome-wide CRISPR KO screen was performed in 501Mel cells, which underwent transduction with a pooled lentiviral library of single guide RNAs (sgRNAs) targeting 19,114 human coding genes, followed by treatment with tcT3 or a control ASO, and subsequent analysis by next-generation sequencing (NGS). Analysis of integrated sgRNA read counts enabled the identification of 95 candidate activators (including WDR91) and 55 candidate inhibitors, whose knockout was predicted to reduce or enhance tcT3 anti-proliferative activity, respectively.

Following the CRISPR KO screen, a series of RNA interference (using siRNA)-based knockdown experiments were conducted to validate the role of WDR91 as a positive regulator of ASO activity. First, knockdown of WDR91 significantly impaired the anti-proliferative activity of tcT3 in 501Mel cells, supporting a role for WDR91 as a positive regulator of tcT3 activity in this cell line. A similar effect was also observed for the T3 ASO (locked nucleic acid [LNA]-PS version of the TSB), indicating that this role is not restricted to a specific ASO chemistry. Furthermore, in U2OS osteosarcoma and PC9 pulmonary adenocarcinoma cell lines, WDR91 knockdown reduced the activity of an LNA-PS gapmer-like ASO targeting MALAT1, as evidenced by decreased *MALAT1* mRNA degradation, supporting a role for WDR91 in enhancing ASO activity across additional cellular contexts and ASO modalities. However, WDR91 knockdown had no significant impact on splice-switching ASOs that induce exon skipping in the *DMD* pre-mRNA in KM571 (an immortalized human myoblast cell line) cells, indicating that the effect of WDR91 on ASO activity is cell type-dependent. Notably, WDR91 knockdown did not affect total ASO uptake, suggesting that its effect occurs downstream of cellular internalization.

The identification of WDR91 as a positive regulator of ASO activity highlights the potential role of endosomal maturation in controlling productive intracellular ASO delivery. Validation across different ASO modalities (i.e., sequence, chemistry, and design) and multiple cell lines further confirms that WDR91 positively regulates ASO activity, although the precise mechanisms underlying this effect remain to be fully defined. Importantly, these findings also indicate that the impact of WDR91 on ASO activity is context-dependent, as its effect was not observed across all ASO modalities or cellular systems. This context dependence suggests that additional endosomal or trafficking factors may influence ASO release and activity, warranting further investigation. Looking forward, these findings provide a foundation for developing strategies that exploit WDR91 or related endosomal regulators to enhance ASO potency in diverse cellular contexts, potentially guiding the design of more effective ASO therapeutics.

## Declaration of interests

We herein declare that there is no existing conflicts of interest for the manuscript entitled “WDR91, an Endosomal Maturation Protein, Promotes Antisense Oligonucleotide Activity.”
